# Research progress on protein tyrosine phosphatase A from Mycobacterium tuberculosis

**DOI:** 10.3389/fimmu.2025.1754992

**Published:** 2026-01-12

**Authors:** Yifei Cai, Leiliang Zhang

**Affiliations:** 1 Department of Clinical Laboratory Medicine, The First Affiliated Hospital of Shandong First Medical University and Shandong Provincial Qianfoshan Hospital, Jinan, Shandong, China; 2 Department of Pathogen Biology, School of Clinical and Basic Medical Sciences, Shandong First Medical University and Shandong Academy of Medical Sciences, Jinan, Shandong, China

**Keywords:** apoptosis, ferroptosis, inhibitor, mycobacterium tuberculosis, PtpA

## Abstract

Mycobacterium tuberculosis (Mtb) protein tyrosine phosphatase A (PtpA) is a crucial tyrosine phosphatase involved in the pathogenesis of tuberculosis. Structural analyses reveal that the W-loop and conserved cysteine residues are essential for the catalytic activity of PtpA, with modifications induced by reactive oxygen species playing a significant role in its function. PtpA suppresses key cellular processes, including phagosome-lysosome fusion and host cell apoptosis, while promoting ferroptosis and disrupting cytokine production to evade host immune responses. Its activity is enhanced by various post-translational modifications, including ubiquitination and phosphorylation, which facilitate its interactions with key cellular pathways. Recent research has identified several selective inhibitors that present promising therapeutic avenues against drug-resistant tuberculosis. This review synthesizes current knowledge on the characteristics, functions, and potential inhibitors of PtpA, underscoring its significance as a therapeutic target in the ongoing battle against tuberculosis and its associated challenges.

## 1 Introduction

Tuberculosis (TB), caused by *Mycobacterium tuberculosis* (Mtb), is one of the most serious infectious diseases in the world. Although it is preventable and curable, it still resulted in 1.25 million deaths in 2023 (1). The primary host cells targeted by Mtb are macrophages, which provide essential nutrients for its growth. This bacterium employs several key virulence mechanisms, including evading membrane fusion between the phagosome and lysosome, suppressing macrophage apoptosis, and establishing a protected intracellular niche. Additionally, it inhibits cytokine production and promotes ferroptosis to enhance its survival and facilitate further spread. Mycobacteria are known to secrete low-molecular-weight tyrosine phosphatases during infection, particularly protein tyrosine phosphatase A (PtpA), which exhibits both tyrosine phosphatase activity and non-enzymatic functions (2). This review will integrate knowledge on the structure and function of PtpA, focusing on its potential as a drug target in addressing the growing challenge of tuberculosis.

## 2 Characteristics of Mtb PtpA

Mtb PtpA is a low molecular weight tyrosine phosphatase. Its expression is induced when mycobacteria growth reaches the stationary phase and remains elevated during the infection of human monocytes (3). Teitelbaum et al. discovered that mycobacteria permeabilize vacuolar membranes (4), and Sullivan et al. found that the SecA2 system is a crucial protein export mechanism necessary for survival within host cells (5). These findings suggest that Mtb is likely to transport PtpA via the SecA2 system and membrane permeabilization. Structural analyses of PtpA have been conducted in two studies. The W-loop, in coordination with the P-loop and D-loop, plays a critical role in shaping and dynamically regulating the substrate-binding pocket, directly participating in substrate-specific recognition and the catalytic process (6).

Research has highlighted key sites of PtpA (**Table 1**). Notably, the conserved cysteine 11 residue, located adjacent to the N-terminus, is crucial for its catalytic function (7). It was further demonstrated that high concentrations of reactive oxygen species (ROS) can lead to the oxidation of the catalytic cysteine (C11), resulting in either temporary or permanent inhibition of PtpA (8). Experimental studies have utilized C11S and C11A mutants (9, 10). Additionally, the Q75L mutant enhances the activity of PtpA by inducing a series of events that relocates the acid loop over the active site and optimally orients the catalytic aspartic acid (D126), facilitating hydrolysis of the thiophosphoryl intermediate (11).

**Table 1:** Functional activities and host targets of PtpA.

Activity	Targets	Mechanism of action	Effects	Mutant	Reference
Inhibition of membrane fusion	Not reported	Dephosphorylation	PtpA promotes the increase of F-actin around phagosomes	C11S	(9)
	H subunit of V-ATPase	Direct binding	PtpA prevents the acidification of phagosomes and enables precise targeting to VPS33B.	L146A, D126A	(22)
	VPS33B	Dephosphorylation	PtpA inhibits phagosome-lysosome fusion.	D126A	(10)
Apoptosis	TRIM27	Direct binding	The competitive binding of PtpA to the RING domain of TRIM27 hinders the latter’s ability to trigger the JNK/p38 MAPK pathway.	D126A	(33)
	Jnk/p38	Dephosphorylation	PtpA inhibits JNK/p38 MAPK pathway.	D126A	(12)
	GSK3α	Dephosphorylation	PtpA dephosphorylates Tyr279 of GSK3α.	Not reported	(29)
Lipid acquisition	TFP	Dephosphorylation	PtpA inhibits β-oxidation of long-chain fatty acids.	D126A, C11S	(34, 35)
Inhibition of cytokine production	TAB3	Direct binding	PtpA associates with TAB3, thus suppressing NF-κB signaling.	D126A	(12)
	GADD45A	Direct binding	PtpA enters the nucleus to regulate the expression of host genes including GADD45A.	D126A	(39)
Ferroptosis	PRMT6	Direct binding	PtpA enters the host cell nucleus and subsequently targets PRMT6 to promote the asymmetric dimethylation of H3R2me2a, thereby inhibiting the expression of GPX4.	C11A, D126A	(42)

Several activators of PtpA have been identified. Ubiquitin has been shown to activate PtpA in the dephosphorylation of phosphorylated vacuolar protein sorting 33B (VPS33B), phosphorylated c-Jun N-terminal kinase (p-JNK), and p-p38 (12). Moreover, the activity of PtpA is enhanced through the phosphorylation of its tyrosine residues 128 and 129 by protein tyrosine kinase A (PtkA) (13–16) and the threonine residue 45 by protein kinase A (PknA) (13). The interaction between PtkA and PtpA warrants further investigation. The structure of PtkA has been analyzed using both nuclear magnetic resonance (NMR) (14) and homology modeling (15). These studies propose that PtkA consists of two domains: the N-terminal highly flexible intrinsically disordered domain (IDD_PtkA_) and the C-terminal rigid kinase core domain (KCD_PtkA_). The highly dynamic nature of IDD_PtkA_ suggests it may employ a fly-casting-like mechanism to regulate its active site in conjunction with KCDP_tkA_ (14, 15). Additionally, two inhibitors of the IDD-KCD interaction, esculin and inosine pranobex, were proposed (15).

The structure of the interaction site of PtpA was investigated in 2012. PtpA features a phosphate-binding loop (P-loop) with the sequence CX_5_R, as well as a loop containing a critical aspartic acid residue (D-loop). PtkA can phosphorylate two well-conserved tyrosine residues, typically located in the D-loop, thereby enhancing PtpA’s activity. Notably, both the P-loop and D-loop play significant roles in the interaction between PtpA and PtkA (6). As a phosphatase, PtpA is subject to substrate activation, specifically by p-nitrophenyl phosphate, phosphotyrosine, and phosphoserine (17).

## 3 Functions of PtpA

### 3.1 Inhibition of phagosome-lysosome membrane fusion

While F-actin aggregation is crucial for phagocytosis, endocytic organelles, and vesicular transport (18), it also impedes direct contact between the phagosome and lysosome membranes (19) Therefore, it is necessary to depolymerize F-actin to facilitate membrane fusion (19). However, PtpA can inhibit this important host defense mechanism (9). Castandet et al. observed that macrophages expressing PtpA exhibited a significant increase in F-actin around their phagosomes, which persisted for a longer duration. This effect was not observed with the phosphatase-inactive mutant C11S (**Table 1**) (9). Additionally, PtpA was shown to impair the phagocytosis of mycobacteria and opsonized zymosan, but had no effect on the phagocytosis of IgG-coated particles (9).

**Figure 1:**
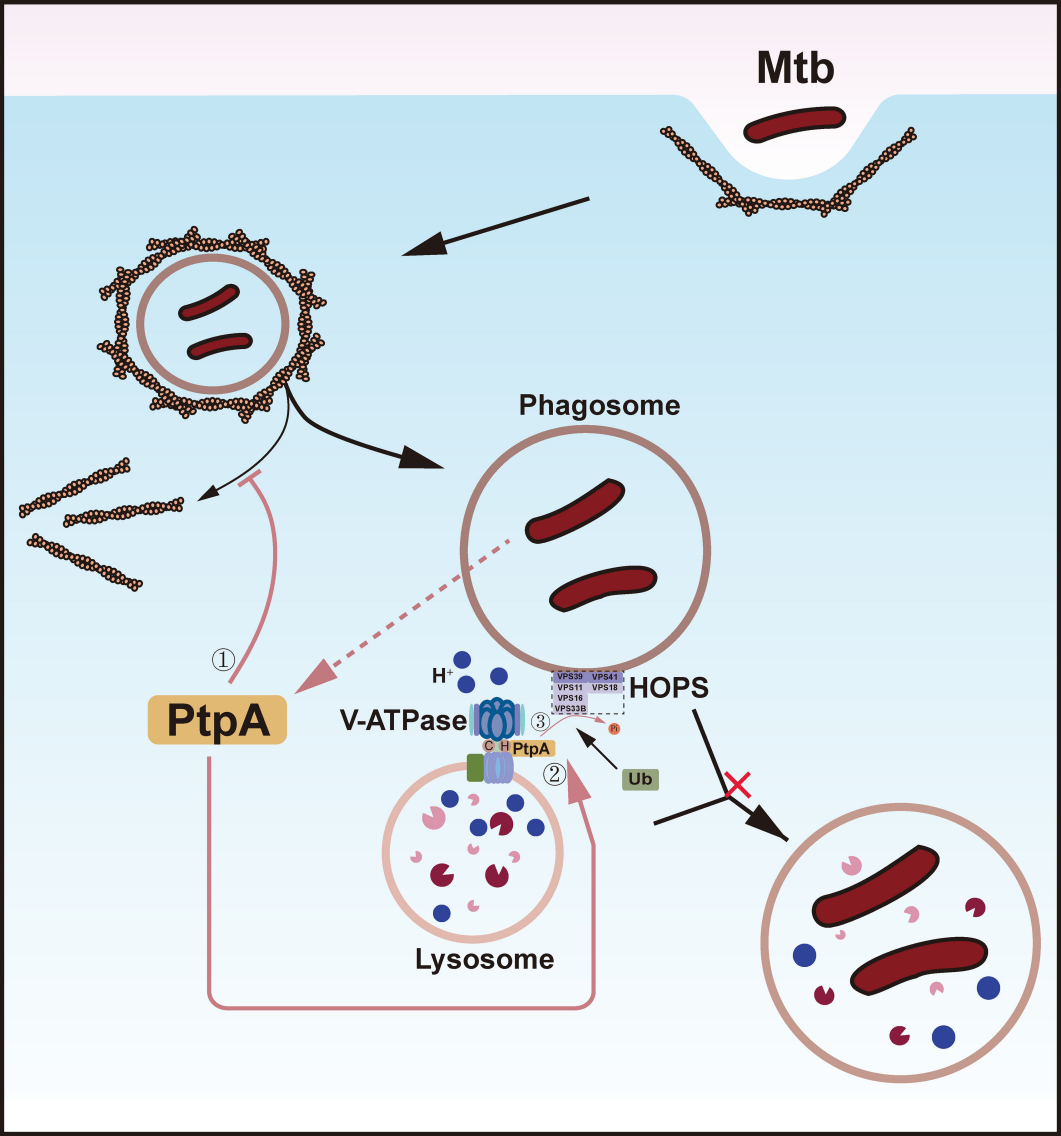
PtpA inhibits phagosome-lysosome membrane fusion. Protein-tyrosine phosphatase A (PtpA), secreted by *Mycobacterium tuberculosis* (Mtb), influences membrane fusion through three mechanisms: 1, PtpA inhibits actin depolymerization, thereby preventing the phagosome from approaching the lysosome. 2, The binding of PtpA to subunit H of the vacuolar H^+^-ATPase (V-ATPase) obstructs its recruitment to the phagosome, which in turn hinders phagosome maturation. Additionally, this binding helps PtpA localize to its catalytic substrate, vacuolar protein sorting 33B (VPS33B). 3, PtpA dephosphorylates VPS33B, which effectively blocks phagosome-lysosome membrane fusion.

The fusion between the mature phagosome and lysosome is mediated by soluble N-ethylmaleimide-sensitive factor attachment protein receptor (SNARE) proteins, with VPS33B acting as a subunit of Class C that is crucial for SNARE-mediated membrane fusion (20). During Mtb infection, it has been reported that PtpA binds to active, phosphorylated VPS33B and dephosphorylates it, thus inhibiting phagosome-lysosome fusion (**Figure 1**) (10).

The phagosome recruits a protein pump called V-ATPase, which utilizes energy from ATP to pump protons inward, rapidly acidifying itself. This process inhibits bacterial growth, enhances antimicrobial enzyme activity, and guides the phagosome to fuse with lysosomes, ultimately destroying invaders and preparing for an immune response (21). Based on these findings, Wong et al. discovered that the binding of PtpA to the H subunit of V-ATPase inhibits its recruitment (22). They also elucidated the dephosphorylation mechanism concerning VPS33B. By associating with distinct accessory subunits, the Class C VPS complex forms either class C core endosomal vacuole tethering (CORVET) or homotypic fusion and vacuole protein sorting (HOPS) complex. CORVET (containing VPS3/VPS8) governs early-to-late endosome fusion, while HOPS (containing VPS39/VPS41) is responsible for lysosomal fusion events (23). The researchers found that V-ATPase may specifically interact with the HOPS complex during phagosome-lysosome fusion (22). Therefore, the newly identified binding of PtpA to the H subunit of V-ATPase not only prevents the acidification of phagosomes but also enables precise targeting to the HOPS complex (possibly involving VPS39, VPS41, and VPS33B), with VPS33B confirmed to be dephosphorylated by PtpA. Consequently, CORVET-mediated early endosome fusions with the phagosome remain intact (22, 24). Activated Rab7 is recognized as a binding partner for HOPS-specific subunits in endosomal maturation (25). Therefore, Wong et al. hypothesized that Rab7’s interaction with HOPS likely functions upstream to initiate the association between V-ATPase and the Class C VPS complex (**Figure 1**) (22).

### 3.2 Inhibition of apoptosis

The ability of Mtb to inhibit apoptosis is critical for the pathogenesis of Mtb, as the death of the host cell would disrupt its specialized niche (26, 27). The activity of glycogen synthase kinase 3 alpha (GSK3α) through tyrosine phosphorylation (Tyr279) is essential for initiating apoptosis (28). To ensure its survival, PtpA from Mtb acts as a phosphatase, dephosphorylating Tyr279 of GSK3α and thus inhibiting host cell apoptosis (**Table 1**) (29).

**Figure 2:**
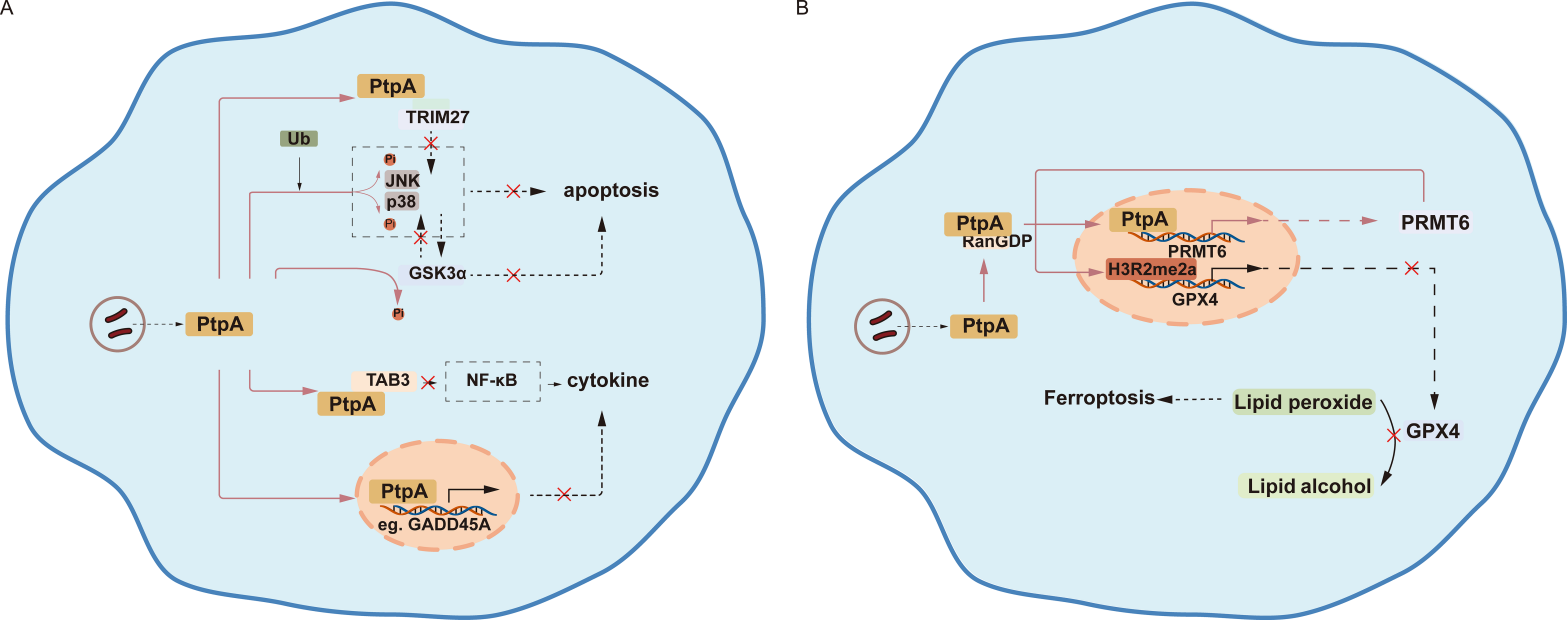
PtpA inhibits apoptosis and cytokine production, while promoting ferroptosis. **(A)** PtpA directly dephosphorylates phosphorylated c-Jun N-terminal kinase (p-JNK) and p-p38 proteins, inhibiting apoptosis activated by ubiquitin (Ub). It also binds to the really interesting new gene (RING) domain of tripartite motif containing 27 (TRIM27), facilitating the dephosphorylation of glycogen synthase kinase 3 alpha (GSK3α), either directly or indirectly influencing apoptotic processes. Furthermore, PtpA’s interaction with transforming growth factor protein beta activated kinase 1 binding protein 3 (TAB3) impedes the nuclear factor kappa B (NF-κB) pathway, thereby suppressing cytokine production. PtpA can also enter the nucleus, where it influences the transcription of proteins related to cytokine production, such as growth arrest and DNA damage-inducible 45 alpha (GADD45A). **(B)** PtpA enters the nucleus in association with RanGDP and enhances the expression of protein arginine methyltransferase 6 (PRMT6), which catalyzes the asymmetric dimethylation of histone H3 at arginine 2 (H3R2me2a) in the glutathione peroxidase 4 (GPX4)-coding region. This modification leads to a decrease in GPX4 expression, ultimately resulting in the accumulation of lipid peroxides and promoting ferroptosis.

The JNK and p38 pathways are among the most important mechanisms inducing apoptosis. It has been reported that PtpA can dephosphorylate JNK and p38, thereby suppressing apoptosis (12). Tripartite motif containing 27 (TRIM27) is essential for the promotion of apoptosis (30). Specifically, TRIM27 possesses a really interesting new gene (RING) domain and functions as an E3 ubiquitin ligase with SUMO E3 ligase activity (31). Apoptosis is enhanced through a mechanism where TRIM27-mediated ubiquitination of USP7 inhibits its deubiquitinating function, leading to the accumulation of ubiquitinated RIP1 (32). Based on these investigations, Wang et al. concluded that the competitive binding of Mtb PtpA to the RING domain of TRIM27 hinders the latter’s ability to trigger the JNK/p38 MAPK pathway and subsequent apoptosis (**Figure 2A**) (33).

### 3.3 Lipid acquisition

PtpA has been reported to specifically target Tyr271 of mitochondrial trifunctional protein (TFP) and dephosphorylate it (34, 35). This residue is located in helix-10 of hTFPα, a region known to be critical for its localization to the mitochondrial membrane and its enzymatic activity (34, 35). This is important because lipids are the primary nutrient source for Mtb (36), and TFP is essential for the β-oxidation of long-chain fatty acids, a process that generates electrons for mitochondrial ATP production (37).

### 3.4 Inhibition of cytokine production and promotion of ferroptosis

Upon encountering pathogens or damage signals, macrophages release a variety of cytokines that act as chemical messengers to activate other immune cells, recruiting them to the site of infection to eliminate threats. The nuclear factor kappa B (NF-κB) pathway is among the most important responses in macrophages. Therefore, PtpA competes for binding to the ubiquitin-interacting region of transforming growth factor protein beta activated kinase 1 binding protein 3 (TAB3), inhibiting the interaction between the host adaptor TAB3, thus suppressing NF-κB signaling and cytokine production (12).

Growth arrest and DNA damage-inducible 45 alpha (GADD45A) is a protein involved in various key cellular processes such as cell division, apoptosis, senescence, and DNA damage repair, influencing the regulation of cell proliferation and migration (38). Wang et al. found that PtpA not only dephosphorylates multiple proteins in the host cytoplasm (including p-JNK, p-p38, and p-VPS33B) but also enters the nucleus to regulate host gene expression (e.g., GADD45A), thus suppressing the host’s innate immune response (**Figure 2A**). Additionally, the N-terminal region of PtpA (amino acids 1–20) plays a critical role in its ability to bind DNA (39).

Cell death modalities can serve opposing roles: they may enhance the host’s defenses against pathogens or be exploited by pathogens for pathogenicity or dissemination, such as ferroptosis (40, 41). It is widely recognized that glutathione peroxidase 4 (GPX4) can catalyze the reaction between GSH and peroxides to inhibit ferroptosis. In Mtb-induced ferroptosis, there is a characteristic depletion of GSH and inhibition of GPX4 (40). Another important point is that protein arginine methyltransferase 6 (PRMT6) is the primary methyltransferase responsible for catalyzing asymmetric dimethylation of histone H3 at arginine 2 (H3R2me2a), a mark of transcriptional inhibition (109–110). In 2023, Qiang et al. discovered that PtpA promotes ferroptosis for pathogenicity and dissemination by interacting with RanGDP to enter the host cell nucleus and subsequently targeting PRMT6 to promote the asymmetric dimethylation of H3R2me2a, thereby inhibiting the expression of GPX4 (**Figure 2B**) (42).

## 4 Inhibitors of PtpA

In 2009, Rawls et al. developed the first inhibitor of PtpA, compound 38, which is highly selective and operates at micromolar levels through hydrogen bonding between DFMP and the active site, as well as π-stacking interactions between the aromatic ring and Trp48 (**Table 2**) (43). The following year, Mascarello et al. identified five synthetic chalcone inhibitors (44) based on an article from 2008 (45). The inhibitory activity of these compounds is primarily governed by two structural features: first, the placement of two methoxyl groups on the A-ring enables the formation of hydrogen bonds with key residues (Arg17, His49, and Thr12) in the PtpA active site. Second, replacing the phenyl ring with a 2-naphthyl group at the B-ring facilitates a hydrophobic π-stacking interaction with Trp48 (44).

**Table 2:** Summary of PtpA inhibitors.

Inhibitors	PtpA K_i_ (μM)	Mechanism	Reference
DFMP compound 38	1.4 ± 0.3	Reversible competitive inhibition	(43)
Synthetic chalcone 4a	21.3 ± 2.6	Reversible competitive inhibition	(44)
Synthetic chalcone 5j	4.9 ± 1.0	Reversible competitive inhibition	(44)
Synthetic chalcone 5a	5.4 ± 1.4	Reversible competitive inhibition	(44)
Synthetic chalcone 5i	17.9 ± 2.9	Reversible competitive inhibition	(44)
Synthetic chalcone 4d	9.1 ± 1.6	Reversible competitive inhibition	(44)
Nitric oxide		S-nitrosylation of the non-catalytic C53	(46)
Thiosemicarbazone compounds 5	1.2	Allosteric inhibition	(47)
Thiosemicarbazone compounds 9	5.6	Allosteric inhibition	(47)
Thiosemicarbazone compounds 18	1.7	Allosteric inhibition	(47)
Cis-2-eicosenoic acid	8.2^a^	Reversible competitive inhibition	(48)
Trans-2-eicosenoic acid	11.26^a^	Reversible competitive inhibition	(48)
Shikonin	8.5	Allosteric inhibition	(49)
Juglone	12.5	Allosteric inhibition	(49)

a IC_50_ value.

In 2012, Matiollo et al. discovered that S-nitrosylation of the non-catalytic C53 (a single cysteine residue) significantly reduces V_max_, without affecting K_m_ (**Table 2**) (46). In 2018, Sens et al. identified thiosemicarbazone compounds 5, 9, and 18 as allosteric inhibitors of PtpA (47). This allosteric site, referred to as the adjacent site, is formed by the residues Ala94, Arg98, Val107, Arg108, Met109, Ser112, Phe113, Pro115, His120, Leu122, Gly148, and Trp152. It is separated from the catalytic site by the flexible D-loop (comprising residues Arg111 to Asp131). The Ki values for these compounds range from 1.2 to 5.6 µM (**Table 2**) (47). In 2020, Savalas et al. filled the gap in natural inhibitors by identifying two fatty acids, cis-2 and trans-2-eicosenoic acids, which demonstrated IC_50_ values at low micromolar concentrations and are prospective candidates for inhibiting latent Mtb (**Table 2**) (48). Their interaction sites are located near the active sites. The binding site of cis-2-eicosenoic acid involves four amino acid residues of PtpA: Asn14, Ile15, Cys16, and Arg17. In contrast, the binding site of trans-2-eicosenoic acid includes two potential amino acid residues: Thr12 and Gly14, with a lesser degree of interaction with Arg17 (48). Further advancements in the field of natural inhibitors occurred in 2023 when Sulyman et al. discovered that both shikonin (5, 8-dihydroxy-2-[(1R)-1-hydroxy-4-methyl-3-pentenyl]-1,4-naphthoquinone) and juglone (5-hydroxy-1,4-naphthalenedione) exhibit strong allosteric inhibition (**Table 2**) (49). Both compounds are classified as naphthoquinones; however, their binding sites still require further investigation.

## 5 Conclusions and prospects

In this review, we emphasize the functions of PtpA, including the inhibition of phagosome-lysosome membrane fusion and host cell apoptosis, acquisition of lipids from the host, suppression of immune responses, and promotion of ferroptosis (**Table 1**). With the increasing understanding of the specific mechanisms involved, numerous studies have suggested that developing inhibitors that specifically target PtpA is a promising strategy for combating tuberculosis (**Table 2**).

Before assessing the feasibility of its inhibitors for treating tuberculosis, it is essential to clearly understand the specific primary pathways that PtpA influences in host cells. Firstly, it can inhibit phagosome membrane fusion through three pathways: by promoting F-actin aggregation to impair direct contact, dephosphorylating VPS33B, and interacting with the H subunit of V-ATPase to hinder acidification. Secondly, it suppresses host cell apoptosis by dephosphorylating GSK3α, associating with ubiquitin to dephosphorylate JNK and p38, and targeting TRIM27. Thirdly, it conserves host lipids by dephosphorylating hTFP, thereby altering macrophage metabolism. Additionally, PtpA suppresses immune responses through its interaction with ubiquitin to prevent TAB3 from undergoing ubiquitination or by inhibiting the transcription of GADD45A. Interestingly, it can also inhibit the expression of GPX4 to promote ferroptosis for pathogenicity and dissemination. How does PtpA achieve these mechanisms? On one hand, it exhibits its tyrosine phosphatase activity; on the other, it directly interacts with certain proteins or genes due to its biological structure.

Homologous proteins, which share a common evolutionary origin, are fundamental to understanding protein function and evolution. PtpA from Mtb has three main homologous counterparts: PtpA from *Mycobacterium bovis* (*M. bovis*), *Mycobacterium avium subspecies paratuberculosis* (*M. paratuberculosis*), and *Mycobacterium marinum* (*M. marinum*) (2). It has been identified that PtpA from *M. paratuberculosis* possesses similar dephosphorylating activity and a secretory effect as seen in Mtb (2) and is a crucial protein for infection detection (50). PtpA from *M. marinum* can also inhibit phagosome-lysosome membrane fusion (51).

However, a significant number of studies have only conducted *in vitro* assays, while *ex vivo* and animal experiments are relatively scarce. Whether certain processes accurately reflect the functioning of the phosphatase still requires further validation through dephosphorylation assays, as relying solely on phosphatase active-site mutants is insufficient.

With an increasing understanding of PtpA’s functions, inhibitors targeting it to combat tuberculosis have gradually emerged. These inhibitors can be categorized into three main types: reversible competitive inhibition, allosteric inhibition, and chemically modifying inhibition. Undoubtedly, the development of new drugs targeting PtpA represents a key breakthrough in the fight against drug-resistant tuberculosis.
